# Comparing the efficacy of regorafenib and 5-fluorouracil-based rechallenge chemotherapy in the third-line treatment of metastatic colorectal cancer

**DOI:** 10.1186/s12885-023-11783-5

**Published:** 2024-01-02

**Authors:** Elif Şenocak Taşçı, Başak Oyan, Özlem Sönmez, Arda Ulaş Mutlu, Muhammed Mustafa Atcı, Abdullah Sakin, İrem Öner, Havva Yeşil Çınkır, Melek Karakurt Eryılmaz, Dilek Çağlayan, Onur Yazdan Balçık, Nail Paksoy, Senem Karabulut, Derya Kıvrak Salim, Cemil Bilir, Miraç Özen, Melike Özçelik, Ali Arıcan, Baran Akagündüz, Ali İnal, Dinçer Aydın, Leyla Özer, Ahmet Gülmez, Nazım Serdar Turhal, Selin Aktürk Esen, Efnan Algın, Sinem Akbaş, Yakup İriağaç, Teoman Şakalar, Çağlar Ünal, Özlem Er, Şaban Seçmeler, Mustafa Bozkurt

**Affiliations:** 1 Department of Medical Oncology, Saglık Bilimleri University, Kanuni Sultan Süleyman Research and Training Hospital, Istanbul, Turkey; 2 Department of Medicine, Acıbadem MAA University, Istanbul, Turkey; 3 Department of Medical Oncology, Haseki Education and Research Hospital, Istanbul, Turkey; 4 Department of Medical Oncology, Medipol Bahçelievler Hospital, Istanbul, Turkey; 5 Department of Medical Oncology, Konya City Hospital, Konya, Turkey; 6 Department of Medical Oncology, Faculty of Medicine, Gaziantep University, Gaziantep, Turkey; 7 Meram Faculty of Medicine, Department of Medical Oncology, Necmettin Erbakan University, Konya, Turkey; 8 Department of Medical Oncology, Mardin Education and Research Hospital, Mardin, Turkey; 9 Department of Medical Oncology, Tekirdağ Dr. İsmail Fehmi Cumalıoğlu City Hospital, Tekirdağ, Turkey; 10 Department of Medical Oncology, Şişli Kolan Hospital, Istanbul, Turkey; 11 Department of Medical Oncology, Antalya Education and Research Hospital, Antalya, Turkey; 12 Department of Medical Oncology, Medical Park Hospital, Istanbul, Turkey; 13 Department of Medical Oncology, Sakarya University Research and Education Hospital, Sakarya, Turkey; 14 Department of Medical Oncology, University of Health Sciences Umraniye Education and Research Hospital, Istanbul, Turkey; 15 Department of Medical Oncology, Binali Yıldırım University, Erzincan, Turkey; 16 Department of Medical Oncology, Mersin City Hospital, Mersin, Turkey; 17 Department of Medical Oncology, Derince Education and Research Hospital, Kocaeli, Turkey; 18 Department of Medical Oncology, Adana City Hospital, Adana, Turkey; 19 Department of Medical Oncology, Anadolu Medical Center, Kocaeli, Turkey; 20 Department of Medical Oncology, Ankara City Hospital, Ankara, Turkey; 21 Department of Medical Oncology, Koç University Hospital, Istanbul, Turkey; 22 Department of Medical Oncology, Namık Kemal University, Tekirdağ, Turkey; 23 Department of Medical Oncology, Necip Fazıl City Hospital, Kahramanmaraş, Turkey; 24 Department of Medical Oncology, Bilim University, Istanbul, Turkey; 25 Department of Medical Oncology, Medical Park Bahçelievler Hospital, Istanbul, Turkey

**Keywords:** Colon cancer, Fluouracil, Rechallenge, Regorafenib, Survival, Third-line treatment

## Abstract

**Background:**

The optimal treatment for metastatic colorectal cancer (mCRC) after the second line is still controversial. Regorafenib has been the standard of care in this setting as it improved overall survival (OS) compared to placebo. In real-world practice chemotherapy rechallenge is also a preferred option even though supporting evidence is not enough. We aim to compare the efficacy of regorafenib and 5-fluorouracil-based (5-FU) rechallenge treatment in the third line setting of mCRC.

**Methods:**

In this retrospective multi-institutional trial, mCRC patients from 21 oncology centers who progressed after 2 lines of chemotherapy were analyzed. Patients who were treated with regorafenib or rechallenge therapy in the third-line setting were eligible. Rechallenge chemotherapy was identified as the re-use of the 5-FU based regimen which was administered in one of the previous treatment lines. OS, disease control rate (DCR), progression free survival (PFS) and toxicity were analyzed.

**Results:**

Three hundred ninety-four mCRC patients were included in the study. 128 (32.5%) were in the rechallenge, and 266 (67.5%) were in the regorafenib group. Median PFS was 5.82 months in rechallenge and 4 months in regorafenib arms (hazard ratio:1.45,95% CI, *p* = 0.167). DCR was higher in the rechallenge group than regorafenib (77% vs 49.5%, respectively, *p* =  < 0.001). Median OS after the third-line treatment was 11.99 (95% CI, 9.49–14.49) and 8.08 months (95% CI, 6.88–9.29) for rechallenge and regorafenib groups, respectively (hazard ratio:1.51, 95% CI, *p* < 0.001). More adverse effects and discontinuation were seen with regorafenib treatment.

**Conclusion:**

Our study revealed that higher disease control and OS rates were achieved with rechallenge treatment compared to regorafenib, especially in patients who achieved disease control in one of the first two lines of therapy.

## Background

Colorectal cancer (CRC) contributes highly to cancer-mortality [1]. De novo metastatic patients compose one fourth of CRC patients where 25–30% of patients with early-stage or locally advanced disease become metastatic within 5 years [2]. The 5-year survival is only 6% [3]. Fluoropyrimidine-based treatments either with oxaliplatin or irinotecan formed the backbone of metastatic CRC (mCRC) for decades [4, 5]. Introduction of targeted therapies to backbone treatment prolonged the overall survival (OS) up to 30 months [6, 7]. Yet, disease progression is inevitable. Once the tumor cells develop resistance to two main cytotoxic drugs used consecutively, the remaining options are very few [8].

The recommended third-line treatments for mCRC are either a multi-targeted tyrosine kinase inhibitor, regorafenib, or oral nucleoside analogue, trifluridine/tipiracil (TAS-102) [8]. While these agents brought an alternative to best supportive care, which was the optimal treatment before, they yield minimal response rate and short progression free survival (PFS) (1.9 months for regorafenib (HR 0.49) and 2 months for trifluridine/tipiracil (HR 0.48)) and OS (6.4 months for regorafenib (HR 0.77) and 7.1 months for trifluridine/tipiracil (HR 0.68) [9, 10]. The limited efficacy of these treatments caused the introduction of chemotherapy rechallenge in clinical practice though there is limited data to support this approach [11–14]. Chemotherapy (CT) rechallenge is reintroduction of the same therapy, after an intervening treatment, to which the tumor developed resistance. There are studies conducted with limited number of patients to assess the efficacy of CT rechallenge [15–17]. The promising results were gained with rechallenge of oxaliplatin, irinotecan, bevacizumab and cetuximab; however, neither of them compared the rechallenge chemotherapy with the standard third-line treatment [18–21]. There are only two retrospective studies comparing regorafenib and rechallenge chemotherapy in the third-line which have contradictory results [22, 23]. In this study, we aimed to evaluate the efficacy of regorafenib versus CT rechallenge in the third-line treatment of mCRC.

## Methods

### Study design and patients

The data of patients with mCRC diagnosed between 2010 and 2019 in 21 oncology centers were analyzed, retrospectively. Patients who were treated with 3 or more lines of chemotherapy were included in the study. The demographic and clinical characteristics of the patients, pathological features, mutation status, treatment schedules and response, follow-up data about safety of treatment and survival data were obtained from medical records. Eastern Oncology Group (ECOG) performance status of patients with 0 and 1 are evaluated in the same group in order to avoid conflict in retrospective data-based studies. Patients were grouped as either rechallenge or regorafenib according to the third-line treatment they received. Oligometastatic disease was defined as up to 5 lesions.

Rechallenge chemotherapy was defined as re-use of the same or the 5-fluouracil based regimen that was administered in one of the first two lines of mCRC. 5-fluouracil (5-FU) based chemotherapy either with oxaliplatin (FOLFOX/XELOX) or irinotecan (FOLFIRI/XELIRI) or both (FOLFIRINOX/XELIRINOX) was administered as rechallenge. Patients were allowed to receive biological treatment consisting of either anti-EGFR antibody (cetuximab, panitumumab) or anti-VEGF antibody (bevacizumab, aflibercept) in combination with chemotherapy according to mutation status of KRAS, NRAS and BRAF. If patients received maintenance treatment after achieving disease control, induction followed by maintenance treatment was considered as one line of therapy. Treatment response evaluation was done according to RECIST 1.1 criteria. Clinical benefit rate (complete response plus partial response plus stable disease) was defined as response to previous line of therapies. The primary end point was OS, and secondary end points were PFS and safety of rechallenge and regorafenib treatment. Subgroup analyses were done to evaluate factors affecting survival.

### Statistical analyses

Data were analyzed using SPSS (Statistical Package for the Social Sciences) version 25.0 (IBM Corp., Armonk, NY, USA). Descriptive statistical methods (number, percentage, median, etc.) were used. PFS was defined as the interval between the date of first cycle of new treatment and the date of progression. OS was calculated from the diagnosis until the date of death from any cause or last follow-up. OS after third-line treatment was calculated from the start of third-line treatment until the date of death from any cause or last follow-up. Kaplan–Meier method was used for survival estimates. The effects of various prognostic factors related to tumor and patient characteristics on progression-free and overall survival were compared by log-rank test. The influence of multiple prognostic factors on progression-free and overall survival were investigated using the multivariate Cox regression test. Categorical comparisons between groups were calculated with Chi-Square Tests (Pearson Chi-Square, Continuity Correction, Fisher's Exact Test). The results were evaluated at the 95% confidence interval and two-sided *p* < 0.05 was considered to indicate statistical significance.

## Results

### Patient characteristics

A total of 394 patients were included in the study. Of these, 128 (32.5%) were in the rechallenge, and 266 (67.5%) were in the regorafenib group. The median age of the patients was 57 (range: 25–84) years. 50% of the patients were female. The proportion of female patients in the rechallenge group was higher than in the regorafenib group (50 vs 35.7%, *p* = 0.023). The two groups’ mutation and metastatic status (synchronous or metachronous) were similar. The detailed demographic and molecular characteristics of patients are shown in Table 1. KRAS, NRAS and BRAF mutation tests were evaluated in 94.4%, 84.7% and 59.6 of the patients, respectively. There was no significant difference between two groups in terms of mutation status.

**Table 1:** Baseline Patient Characteristics

		**Rechallenge (*n* = 128) *n*(%)**	**Regorafenib (*n* = 266) n(%)**	***p***
Age, median (min–max)		55 (33–80)	58 (25–84)	*.170*
Sex	Female	64 (50)	95 (37.8)	*.023*
	Male	64 (50)	156 (62.2)	
ECOG PS	0/1	115 (89.8)	228 (85.7)	*.253*
	2	13 (10.2)	38 (14.4)	
Tumor side	Right colon	18 (14.1)	54 (20.4)	*.129*
	Left colon	110 (85.9)	211 (79.6)	
Metastatic status^a^	Metachronous	48 (37.5)	110 (41.4)	*.464*
	Synchronous	80 (62.5)	156 (58.6)	
Metastatic Site^a^	Single Site	88 (73.9)	167 (65.7)	*.112*
	Multiple Sites	31 (26.1)	87 (34.3)	
Liver Only	Yes	71 (55.5)	157 (59.1)	*.503*
	No	57 (44.5)	109 (40.9)	
KRAS	Wildtype	71 (55.4)	145(54.5)	*.337*
	Mutant	44 (34.3)	112 (42.1)	
	Missing	13 (10.1)	9 (3.4)	
NRAS	Wildtype	78 (60.9)	194 (72.9)	*.122*
	Mutant	24 (18.7)	38 (14.2)	
	Missing	26 (20.3)	34 (12.7)	
BRAF	Wildtype	59 (46)	160 (60.1)	*.158*
	Mutant	7 (5.4)	9 (3.4)	
	Missing	62 (48.4)	97 (36.4)	
Microsatellite status	MSS	14 (10.9)	43 (16.1)	*.279*
	MSI-high	2 (1.6)	2 (0.75)	
	Missing	112 (87.5)	221 (83.1)	

*ECOG PS* Eastern Oncology Group Performance Scale, *MSI* microsatellite instability, *MSS* microsatellite stable

^a^Status at the time of diagnosis

### Treatment

In the rechallenge group, 60 patients received oxaliplatin-FU-based chemotherapy like FOLFOX or XELOX (in combination with anti-VEGF treatment in 34 patients, in combination with anti-EGFR treatment in 18 and without a biological agent in 8). 39 patients received FOLFIRI or XELIRI (in combination with anti-VEGF treatment in 25 patients, in combination with anti-EGFR treatment in 10, and without a biological agent in 4). 23 patients received FOLFIRINOX (in combination with anti-VEGF treatment in 17 patients, in combination with anti-EGFR treatment in 2, and without a biological agent in 4). Other treatments were as follows: capecitabine monotherapy (5 patients) and infusion 5-FU/Leucovorin (1 patient). 71 patients (55.5%) in the rechallenge group received the same chemotherapy as the first-line, and 29 (22.6%) patients as the second-line treatment.

In the rechallenge group, 76 (59.3%) patients received anti-VEGF treatment in combination with chemotherapy, whereas 30 patients (23.4%) received anti-EGFR treatment in combination with chemotherapy (Table 2). Regarding biological agents, 73 (68.9%) patients in the rechallenge group were exposed to the same biological agent either in the first- or second line. However, 33 (31.1%) patients received a biological agent that they were not exposed to in either previous line. In 16 patients, prior response to chemotherapy used in rechallenge was progression. Of these, 6 patients received the same biological agent (3 responded as progressive disease, 2 had partial response and 1 had stable disease with the same agent in third line) and 10 patients received different biological agent (3 had stable disease, 1 had partial response and 6 had progressive disease).

**Table 2:** Characteristics of Rechallenge Treatment

	**Rechallenge (*n* = 128)**
**Chemotherapy,** *n* (%)	
Oxaliplatin based treatment (FOLFOX/XELOX)	60 (46.8)
Irinotecan based treatment (FOLFIRI/XELIRI)	39 (30.4)
Irinotecan and oxaliplatin based treatment (FOLFIRINOX)	23 (17.9)
Others^a^	6 (4.6)
**Rechallenge chemotherapy regimen (*n* = 128)**	
Same as first-line, *n* (%)	71 (55.5)
Same as second-line, *n* (%)	29 (22.6)
Both oxaliplatin and irinotecan in rechallenge, *n* (%)	22 (17.2)
Only FU-based, *n* (%)	6 (4.7)
**Biological agent combined with chemotherapy, *n*(%)**	
Anti-VEGF	76 (59.3)
Anti-EGFR	30 (23.4)
None	22 (17.2)
**Prior exposure of the biological agent used in rechallenge, (*n* = 106, %)**	
Yes	73 (68.9)
No	33 (31.1)
Anti-VEGF in third-line	10 (30)
Anti-EGFR (cetuximab/panitumumab) in third-line	23 (70)
**Prior response to chemotherapy used in rechallenge, *n*(%)**	
CR + PR + SD	112 (70.8)
PD	16 (29.1)

*XELOX* capecitabine and oxaliplatin, *CR* complete response, *PR* partial response, *SD* stable disease, *PD* disease progression, *VEGF* vascular endothelial growth factor, *EGFR* endothelial growth factor receptor

^a^Others include capecitabine monotherapy, 5-FU/Leucovorin infusion

The ratio of patients responding to both the first- and second-line of chemotherapy was similar (64.6 vs. 65.6% for the regorafenib and rechallenge group, respectively). In the regorafenib group, 5.1% of patients had progressive disease as the best response to both lines of treatment. At the same time, there were no patients that were resistant to both prior lines of therapy in the rechallenge group (Table 3).

**Table 3:** Response to Prior Lines in Regorafenib and Rechallenge Groups

	**Regorafenib (*n* = 266)**	**Rechallenge (*n* = 128)**	***p***
Response to both prior lines of treatment (CR + PR + SD), *n*(%)	172 (64.6)	84 (65.6)	*.851*
Response to one prior line of treatment (CR + PR + SD), *n*(%)	79 (29.6)	44 (34.3)	*.348*
Progression under both lines of treatment, *n*(%)	15(5.1)	0(0)	*.004*

*CR* complete response, *PR* partial response, *SD* stable disease

43.4% (*n* = 171) of the patients received fourth-line treatment. The rate of forth-line treatment was 58.5% in the rechallenge group and 36% in the regorafenib group (*p* =  < 0.001). 46 patients (35.9%) in the rechallenge group received regorafenib as fourth-line treatment.

### Efficacy

**Fig. 1:**
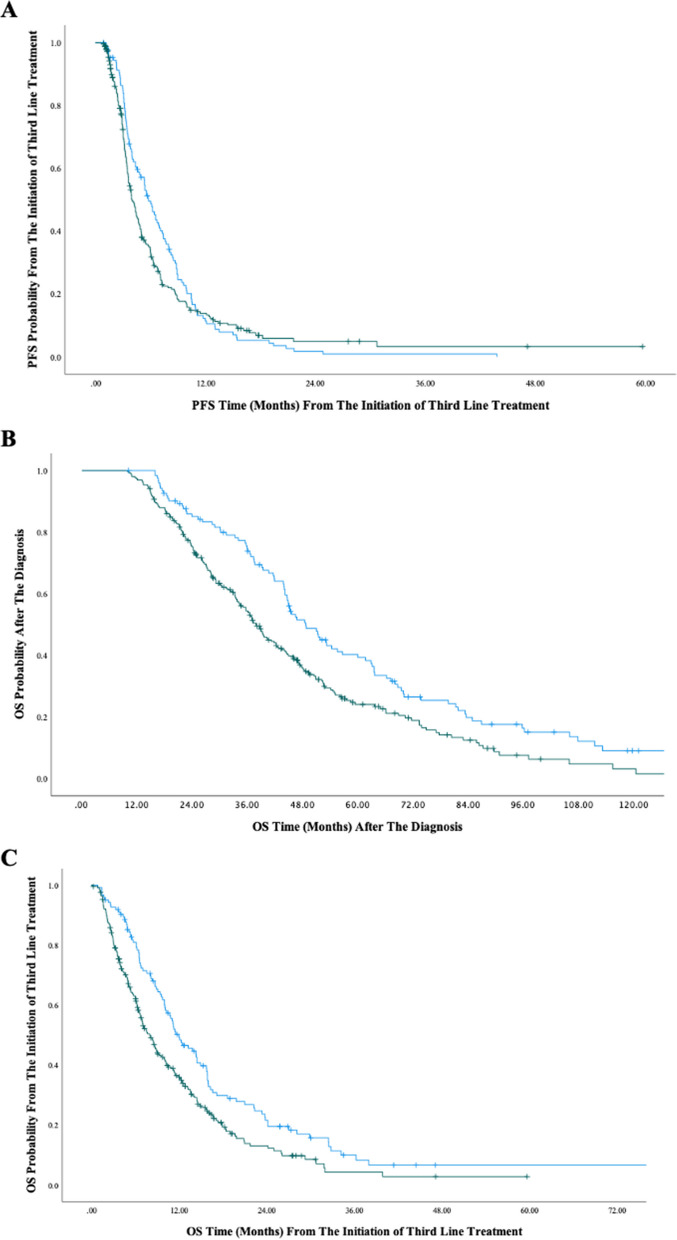
**A** Progression Free Survival after the Initiation of Third-line Treatment. **B** Overall Survival from the Diagnosis. **C** Overall Survival from the Initiation of Third-line Treatment

The median follow-up was 42.55 (range, 39.39–45.70) months from the diagnosis. Median PFS was 10.7 months after first-line, 8.4 months after second-line, and 4.53 months after third-line treatment for all patients. In the third-line treatment, the disease control rate was higher in the rechallenge treatment group than in the regorafenib group (77% vs 49.5%, respectively, *p* =  < 0.001). The median PFS was 5.82 months for rechallenge and 4.01 months for regorafenib group in third-line treatment. There was no statistically significant difference between two groups (*p* = 0.167, Fig. 1A). The univariate analysis revealed that PFS after third-line treatment was significantly longer in women (*p* < 0.001), in patients with metachronous metastasis (*p* = 0.031) (Table 4) and in patients with RAS mutation (*p* = 0.009). Multivariate analysis was performed to assess the independent predictors for PFS. Male sex [HR = 1.52(1.21–1.91), *p* < 0.001] and resistance to both prior lines of treatment [HR = 3.19(1.72–5.94), *p* < 0.001) were independent predictors of shorter PFS.

**Table 4:** Progression Free Survival after Third-line Treatment

		***n***	**Median PFS, months (95%CI)**	***p***
**Treatment**	Rechallenge	128	5.82 (4.97–6.66)	*.167*
	Regorafenib	266	4.01 (3.48–4.53)	
**Age, years**	< 60	226	4.47 (3.85–5.09)	*.555*
	≥ 60	165	4.57 (3.28–5.85)	
**Sex**	Female	159	5.75 (4.59 – 6.91)	< *.001*
	Male	219	4.11 (3.46 – 4.76)	
**ECOG PS**	0/1	267	4.69 (3.95 – 5.44)	*.133*
	2	51	3.94 (2.84–5.08)	
**Tumor side**	Right colon	72	4.37 (3.47 – 5.27)	*.925*
	Left colon	320	4.69 (4.04 – 5.35)	
**Metastases status**^**a**^	Synchronous	236	4.37 (3.56 – 5.09)	.031
	Metachronous	157	4.79 (3.95 – 5.65)	
**Response to prior 2 lines**	Disease control in both lines	256	4.86 (4.24–5.49)	< *.001*
	Disease control in one line	123	4.01 (3.02–4.99)	
	Resistant to both lines	15	2.96 (2.75–3.16)	

*ECOG PS* Eastern Oncology Group Performance Scale

^a^ = at the time of diagnosis

During follow-up, 363 (77.2%) patients died due to disease progression (Table 5). Median OS from diagnosis of metastatic disease was 48.73 months in the rechallenge and 37.95 months in the regorafenib group (*p* = 0.001, Fig. 1B). OS after the third-line treatment was significantly better in the rechallenge group compared to the regorafenib group (11.99 vs 8.08 months, HR:1.51(1.18–1.94), *p* < 0.001, Fig. 1C). OS after third-line of treatment was better in females (*p* = 0.011) and in those who responded to first 2 lines of treatment (*p* < 0.001). Although tumor sidedness (right vs left) and metastasis status (metachronous vs synchronous) were predictive factors for OS after the initial diagnosis of metastasis, these factors did not influence OS after third-line treatment. RAS mutation status was not a predictive factor for OS (*p* = 0.240).

**Table 5:** Overall Survival after Diagnosis and Third-line Treatment

		***n***	**Median Survival from diagnosis, months (95%CI)**	***p***	**Median Survival after third-line, months (95%CI)**	***p***
**Treatment**	Rechallenge	123	48.73 (42.12–55.33)	*.001*	11.99 (9.49–14.49)	< *.001*
	Regorafenib	259	37.95 (34.65–41.24)		8.08 (6.88–9.29)	
**Age, years**	< 60	218	44.49 (39.72–49.25)	*.101*	9.43 (7.86–10.99)	*.557*
	≥ 60	163	40.27 (34.83–45.67)		10.05 (8.27–11.83)	
**Sex**	Female	157	44.91 (43.17–46.64)	*.011*	11.30 (9.09–13.51)	< *.001*
	Male	215	39.33 (36.03–42.26)		8.08 (6.20–9.96)	
**ECOG PS**	0/1	260	42.78 (37.96–47.59)	*.199*	10.58 (8.63–12.52)	*.138*
	2	48	37.65 (24.04–51.26)		6.64 (4.89–8.38)	
**Tumor side**	Right	69	32.33 (23.88–40.78)	*.024*	7.62 (5.67–9.58)	*.074*
	Left	312	44.65 (41.61–47.69)		10.05 (8.66–11.45)	
**Metastases status**^**a**^	Synchronous	229	34.07 (29.72–38.43)	< *0.001*	9.43 (8.12–10.74)	*.164*
	Metachronous	153	57.66 (49.38–65.94)		9.63 (7.17–12.08)	
**Response to first 2 lines**	Disease control in both lines	248	47.08 (43.63–50.53)	< *.001*	10.74 (9.59–11.89)	*.001*
	Disease control in 1 line	119	31.80 (24.37–39.24)		7.56 (5.64–9.47)	
	Resistant to both lines	15	14.92 (11.59–18.23)		3.94 (2.82–5.06)	

*ECOG PS* Eastern Oncology Group Performance Scale

^a^Status at the time of diagnosis

In the multivariate analysis, the independent determinants of longer OS after diagnosis of metastasis were female sex [HR = 0.75 (0.59–0.95), p = 0.023], rechallenge treatment [HR = 0.681 (0.528–0.879), *p* = 0.003] and chemosensitivity to both prior lines of treatment [HR = 0.290 (0.167–0.506), *p* < 0.001]. The independent determinants of longer OS after third-line treatment were female sex [HR = 0.69 (0.54–0.88), *p* = 0.003], rechallenge treatment [HR = 0.717 (0.556–0.924), *p* = 0.010] and response to both prior lines of treatment [HR = 0.428 (0.245–0.748), *p* = 0.003].

There was no statistically significant PFS difference between the rechallenge and regorafenib arms according to the response to previous lines of therapy (Table 6). Median OS was significantly better in rechallenge arm if the patients were sensitive to at least one prior line of therapy (*p* < 0.001 and *p* = 0.031 for both lines and one line, respectively). There was no significant survival difference between patients who received new biological agent during rechallenge and who received the one they were previously exposed to (11.99 and 11.47 months, respectively, *p* = 0.942).

**Table 6:** Progression free and Overall Survival according to Response to Prior Lines of Therapy

	**Rechallenge (*n* = 128)**	**Regorafenib (*n* = 266)**	***p***
**Median PFS**			
Responded to both lines of prior therapy	6.57 (4.95–8.20)	4.3 (3.70–4.9)	*.170*
Responded to one line of prior therapy	4.37 (1.97–6.77)	3.94 (2.77–5.12)	*.763*
Resistant to both		2.96 (2.75–3.16)	
**Median OS after third-line of treatment**			
Responded to both lines of prior therapy	11.99 (9.49–14.49)	8.08 (6.88–9.29)	< *.001*
Responded to one line of prior therapy	8.15 (0.01–17.81)	6.97 (4.62–9.31)	*.031*
Resistant to both		3.94 (2.83–5.06)	

*PFS* progression free survival, *OS* overall survival

### Safety

Adverse events (AE) were observed in 308 (78.2%) patients. Grade 3 and 4 AE were seen in 70 (17.8%) and 9 (2.3%) patients. The incidence of AE in the regorafenib group was statistically higher than the rechallenge group (84.2% vs 65.6%, *p* =  < 0.001). Dose reduction was done in 155 (60.7%) patients in the regorafenib group. 74 (47.7%) of these reductions were 25% of the original dose. In the regorafenib group, patients whose dose were reduced by 50% or more had a shorter OS (6.05 vs 11.21 months respectively, *p* = 0.003). Nausea, diarrhea, mucositis, acneiform rash, and hepatotoxicity were significantly higher in the regorafenib group, while neuropathy was higher in the rechallenge (*p* < 0.001). 25 (10.1%) patients in regorafenib and 3 (4.2%) patients in the rechallenge group discontinued treatment due to side effects. No treatment-related deaths occurred. Adverse events are summarized in Table 7.

**Table 7:** Adverse events

	**Rechallenge (*n* = 128)**		**Regorafenib (*n* = *266*)**		***p***	***p***
	**Any Grade**	**Grade3/4**	**Any Grade**	**Grade3/4**	**Any Grade**	**Grade 3/4**
	**n(%)**					
**Hematological**						
Neutropenia	48 (37.5)	16 (12.5)	83 (31.2)	33 (12.4)	*.214*	*.979*
Thrombocytopenia	37 (28.9)	5 (3.9)	87 (32.7)	10 (3.8)	*.447*	*.943*
Anemia	58 (45.3)	0 (0)	148 (55.6)	9 (3.4)	*.055*	*.034*
**Non-hematological**						
Nausea	55 (43)	3 (2.3)	174 (65.4)	26 (9.8)	< *.001*	*.007*
Diarrhea	37 (28.9)	2 (1.6)	154 (39.1)	21 (7.9)	< *.001*	*.011*
Mucositis	28 (21.9)	0 (0)	141 (53)	17 (6.4)	< *.001*	*.002*
Acneiform rash	7 (5.5)	1 (0.8)	69 (25.9)	10 (3.8)	< *.001*	*.112*
Hepatotoxicity	10 (7.8)	0 (0)	186 (30.1)	5 (1.9)	< *.001*	*.179*
Renal toxicity	12 (9.4)	0 (0)	30 (11.3)	2 (0.8)	*.566*	> *.999*
Neuropathy	55 (43)	8 (6.3)	57 (21.4)	4 (1.5)	< *.001*	*.023*

## Discussion

The most powerful treatment against mCRC is 5-FU based treatments. However, progression after two lines of doublet chemotherapy is unavoidable. Contribution with biological agents has little impact in improvement of OS. In this setting, rechallenge treatment was started to be investigated. Although regorafenib is the standard third-line treatment of mCRC in guidelines, it is still not clear if it is the optimal approach since it was just compared to placebo instead of an active treatment arm [8].

In this study, rechallenge treatment showed better disease control rate and OS compared to regorafenib treatment in third-line. However, the difference in PFS was not statistically significant. The cancer-specific survival data is missing in our study. There was no significant survival difference between patients who received a new biological agent during rechallenge and who received the one they were previously exposed to. So the efficacy of rechallenge treatment can only be attributed to rechallenge chemotherapy. Another possible reason of the lower survival rates seen with regorafenib may be the need for dose reduction related to adverse events. 60% of regorafenib patients required ≥ 50% dose reduction due to intolerance. The decrease in efficacy with inadequate doses of regorafenib is a well-known entity. Regorafenib was also shown to cause more toxicity resulting in discontinuation of treatment. As a result, fourth-line treatment chance is higher with rechallenge treatment. Our study also revealed that chemosensitivity to prior lines of treatment was a major factor in choosing between the treatment options.

The CORRECT and CONCUR trials proved OS improvement with regorafenib against placebo after ≥ 2 or more lines of therapy (1.4 and 2 months, respectively) [9, 24] The PFS and OS with regorafenib treatment in our study (4.01 and 8.08 months, respectively) were comparible with the findings of CONCUR trial (3.2 and 8.8 months, respectively). The higher PFS results in real-world studies can be attributed to the treatment response evaluation intervals (6–8 weeks in CORRECT and CONCUR trial vs 3–4 months in our study group). Main shortcoming of CONCUR and CORRECT trials were comparing regorafenib with placebo instead of active-control. The PFS benefit is probably attributable to the ineffectiveness of control group. Most of the mCRC patients has performance score of 0–1 even after the second-line of treatment and are eligible for systemic treatments. Therefore, placebo would be an under-treatment for this population.

Before the approval of regorafenib in mCRC treatment, few studies questioned the role of chemotherapy rechallenge in third-line. In an explanatory study, promising OS benefit was seen with oxaliplatin rechallenge therapy in patients who had received previous oxaliplatin and irinotecan therapy [25]. The rechallenge therapies assessing the efficacy of irinotecan also reported survival benefit with median OS of 6 and 7.3 months [19, 26]. The limitation of these studies was not using 5-FU as the backbone treatment in the rechallenge. In our study, 95% of the patients in the rechallenge arm received 5-FU based chemotherapy. Although it is not a valid approach to do indirect comparisons, OS rates are better in the literature when irinotecan or oxaliplatin are used in combination with 5-FU derivatives rather than used as a single agent. Our study supports using 5FU based regimens in later lines of treatment.

The effect of rechallenge is well documented but not compared to standardized third-line treatment before 2019. There are three retrospective trials in the literature with controversial results comparing the efficacy of regorafenib with chemotherapy rechallenge in the third-line setting [22, 23]. The PROSERpINA study [17] (*n* = 341), although underpowered and retrospective, showed the survival benefit of rechallenge against regorafenib and TAS-102 in small number of patients. Ergun et al. [22] (*n* = 61) couldn’t find any significant difference between rechallenge and regorafenib treatment in the third-line setting of mCRC in terms of OS and PFS, nevertheless, recommended rechallenge as an alternative treatment option to regorafenib in progressive disease. They showed a better trend for survival with chemotherapy rechallenge if time to rechallenge was more than 6 months, but this also did not reach statistical significance. Kostek et al. [23] (*n* = 104), on the other hand, revealed that rechallenge is more effective than regorafenib in terms of PFS and OS, especially in patients with partial response or stable disease after first- and second-line therapies. These studies constitute the most similar design with rechallenge arm of our study however the main disadvantage of these studies is small sample size which should be cautiously interpreted. The full-text of PROSERpINA study has not been published so we cannot reach the details to make comparisons. Our study had similar characteristic in terms of ethnicity, mean age and performance status compared to other retrospective series. Our study reports the highest number of patients in literature comparing the two treatments and having OS benefit. One of the most likely explanations of OS benefit is the possibility of access to more lines of treatment after rechallenge therapy, whereas most of the patients are not eligible for further treatment after regorafenib treatment. The ongoing randomized FIRE-4 trial [27] is the first prospective trial seeking the answer of rechallenge vs regorafenib in mCRC in the third-line setting. While waiting for the results of FIRE-4 trial, considering the lack of proactive data, the rechallenge therapy is another alternative treatment in the third-line treatment of mCRC.

The adverse events seen with rechallenge and regorafenib may change the course of treatment. In 81 patients, regorafenib dose was reduced by ≥ 50% and this reduction was related to worse survival (6.05 vs 11.21 months, respectively). In a study done by Dane et al. [28], regorafenib related toxicities led to treatment discontinuation in 17% of patients which is compatible with the literature. The most common side effect of chemotherapy rechallenge was neuropathy which may sometimes be handled just with palliative treatment or dose reduction. The poorly tolerated treatments may cause non-compliance, decrease efficacy and cause these patients to lose their opportunity to continue with fourth-line of treatment.

After the introduction of rechallenge in 2015 for the first time, ESMO guidelines mentioned the possibility of reintroducing the same therapy that the tumor has already been exposed to [29]. The mechanism underneath the success of chemotherapy rechallenge is still a question mark. It can be explained by the cellular heterogeneity of the cancer [30]. Tumor harbors cells that are resistant and sensitive to the treatment at the same time. Second line therapies may sensitize patients to the previous therapies by promoting the growth of sensitive clones to first line treatment or epigenetic changes may result in reversable tumor resistance after drug holiday [31].

The major limitation of our study is its retrospective nature. The medical records reached from different centers may host bias. One of the most important data on treatment decisions, ECOG PS, is not fully reliable in data-based studies. Another possible limitation that cause lack of PFS benefit might be biased information regarding the timing of disease progression. This can affect the accuracy of PFS measurements. Another one is the unmeasured confounding variables or factors that influence disease progression but are not considered in the analysis. OS, being a more comprehensive endpoint, accounts for a broader range of factors that contribute to overall survival. Nevertheless, our patient population is representative of daily practice and when the efficacy of regorafenib in patients who progressed after multiple lines of therapy is considered, reintroduction of chemotherapy is a good third-line scenario to increase the treatment options in mCRC until randomized studies are designed or prospective studies are concluded.

## Conclusion

In the absence of data from prospectively randomized trials, comparing the rechallenge treatment with regorafenib in mCRC, our study contributes valuable data to literature. Higher disease control and OS rates were achieved with rechallenge treatment compared to regorafenib, especially in patients who achieved disease control in one of the first two lines of therapy. Rechallenge treatment should be preferred as a valuable option in patients with mCRC in third-line against approved therapies.

## Data Availability

The database of the study is available in the corresponding author and will be sent when requested by e-mail.

## Abbreviations

CRC: Colorectal cancer
mCRC: Metastatic colorectal cancer
PFS: Progression free survival
OS: Overall survival
CT: Chemotherapy
