# Correction: Comparing the efficacy of regorafenib and 5-fluorouracil-based rechallenge chemotherapy in the third-line treatment of metastatic colorectal cancer

**DOI:** 10.1186/s12885-024-11863-0

**Published:** 2024-01-23

**Authors:** Elif Şenocak Taşçı, Başak Oyan, Özlem Sönmez, Arda Ulaş Mutlu, Muhammed Mustafa Atcı, Abdullah Sakin, İrem Öner, Havva Yeşil Çınkır, Melek Karakurt Eryılmaz, Dilek Çağlayan, Onur Yazdan Balçık, Nail Paksoy, Senem Karabulut, Derya Kıvrak Salim, Cemil Bilir, Miraç Özen, Melike Özçelik, Ali Arıcan, Baran Akagündüz, Ali İnal, Dinçer Aydın, Leyla Özer, Ahmet Gülmez, Nazım Serdar Turhal, Selin Aktürk Esen, Efnan Algın, Sinem Akbaş, Yakup İriağaç, Teoman Şakalar, Çağlar Ünal, Özlem Er, Şaban Seçmeler, Mustafa Bozkurt

**Affiliations:** 1 Department of Medical Oncology, Saglık Bilimleri University, Kanuni Sultan Süleyman Research and Training Hospital, Istanbul, Turkey; 2 Department of Medicine, Acıbadem MAA University, Istanbul, Turkey; 3 Department of Medical Oncology, Haseki Education and Research Hospital, Istanbul, Turkey; 4 Department of Medical Oncology, Medipol Bahçelievler Hospital, Istanbul, Turkey; 5 Department of Medical Oncology, Konya City Hospital, Konya, Turkey; 6 Department of Medical Oncology, Faculty of Medicine, Gaziantep University, Gaziantep, Turkey; 7 Meram Faculty of Medicine, Department of Medical Oncology, Necmettin Erbakan University, Konya, Turkey; 8 Department of Medical Oncology, Mardin Education and Research Hospital, Mardin, Turkey; 9 Department of Medical Oncology, Tekirdağ Dr. İsmail Fehmi Cumalıoğlu City Hospital, Tekirdağ, Turkey; 10 Department of Medical Oncology, Şişli Kolan Hospital, Istanbul, Turkey; 11 Department of Medical Oncology, Antalya Education and Research Hospital, Antalya, Turkey; 12 Department of Medical Oncology, Medical Park Hospital, Istanbul, Turkey; 13 Department of Medical Oncology, Sakarya University Research and Education Hospital, Sakarya, Turkey; 14 Department of Medical Oncology, University of Health Sciences Umraniye Education and Research Hospital, Istanbul, Turkey; 15 Department of Medical Oncology, Binali Yıldırım University, Erzincan, Turkey; 16 Department of Medical Oncology, Mersin City Hospital, Mersin, Turkey; 17 Department of Medical Oncology, Derince Education and Research Hospital, Kocaeli, Turkey; 18 Department of Medical Oncology, Adana City Hospital, Adana, Turkey; 19 Department of Medical Oncology, Anadolu Medical Center, Kocaeli, Turkey; 20 Department of Medical Oncology, Ankara City Hospital, Ankara, Turkey; 21 Department of Medical Oncology, Koç University Hospital, Istanbul, Turkey; 22 Department of Medical Oncology, Namık Kemal University, Tekirdağ, Turkey; 23 Department of Medical Oncology, Necip Fazıl City Hospital, Kahramanmaraş, Turkey; 24 Department of Medical Oncology, Bilim University, Istanbul, Turkey; 25 Department of Medical Oncology, Medical Park Bahçelievler Hospital, Istanbul, Turkey

**Correction: BMC Cancer 24, 16 (2024)**

10.1186/s12885-023-11783-5

Following publication of the original article [1], the authors reported an error in the affiliations of authors Başak Oyan, Özlem Sönmez, Leyla Özer, Ali Arıcan, Özlem Er and Mustafa Bozkurt. These authors are all affiliated to institution 2.

The original article [1] has been corrected.
